# Customized depolarization spatial patterns with dynamic retardance functions

**DOI:** 10.1038/s41598-021-88515-x

**Published:** 2021-05-03

**Authors:** David Marco, Guadalupe López-Morales, María del Mar Sánchez-López, Ángel Lizana, Ignacio Moreno, Juan Campos

**Affiliations:** 1 Instituto de Bioingeniería, Universidad Miguel Hernández de Elche, Avda. Universidad s/n, 03202 Elche, Spain; 2 Departamento de Física Aplicada, Universidad Miguel Hernández de Elche, Avda. Universidad s/n, 03202 Elche, Spain; 3 Departamento de Física, Universitat Autònoma de Barcelona, 08193 Bellaterra, Spain; 4 Departamento de Ciencia de Materiales, Óptica y Tecnología Electrónica, Universidad Miguel Hernández de Elche, Avda. Universidad s/n, 03202 Elche, Spain

**Keywords:** Adaptive optics, Displays, Optoelectronic devices and components

## Abstract

In this work we demonstrate customized depolarization spatial patterns by imaging a dynamical time-dependent pixelated retarder. A proof-of-concept of the proposed method is presented, where a liquid–crystal spatial light modulator is used as a spatial retarder that emulates a controlled spatially variant depolarizing sample by addressing a time-dependent phase pattern. We apply an imaging Mueller polarimetric system based on a polarization camera to verify the effective depolarization effect. Experimental validation is provided by temporal integration on the detection system. The effective depolarizance results are fully described within a simple graphical approach which agrees with standard Mueller matrix decomposition methods. The potential of the method is discussed by means of three practical cases, which include non-reported depolarization spatial patterns, including exotic structures as a spirally shaped depolarization pattern.

## Introduction

Controlling the polarization of light is an essential aspect in many different optical techniques^1^, and its detection is the basis of polarimetry and ellipsometry^2^. In recent years there has been a great research activity in developing optical polarimetric instruments, mostly based on liquid–crystal (LC) modulators, capable of generating and/or detecting different states of polarization (SoP), to build polarimeters without moving elements^3–5^.

Partially polarized light can be expressed as a superposition of fully polarized light and unpolarized light weighted by its degree of polarization (DoP). The DoP provides very valuable additional information in the polarimetric analysis of samples^6^, which is becoming especially relevant in biomedical samples where depolarization measurements can provide valuable information^7–9^. The term depolarization refers to the temporal and/or spatial incoherent coupling of polarized light into unpolarized light and is associated with a reduction in the DoP. There are situations where it is convenient to reduce the DoP of fully polarized light. This is typically necessary when a polarization insensitive response is required, as for instance in a laser pump diode^10^, in fluorescence resonance energy transfer (FRET) systems^11^, in laser microfabrication methods^12^ or in optical coherence tomography systems^13^.

There are different techniques to reduce the DoP. A classical method is the Lyot depolarizer^14^, which consists of two wave plates with a 2∶1 thickness ratio and with a 45° relative orientation angle of their optical axes. This device is intended for use with polychromatic light and it has been applied both with bulk optics^14^ and with fiber optics^10^. Other recent techniques apply different strategies, as for instance, dividing the input beam in two orthogonal SoPs which are then recombined after modification of their properties in a Mach-Zenhder interferometer^15,16^, or based on an imbalanced dual-frequency dual-polarization light source^17^.

LC devices and LC modulators have been employed to build optical depolarizers, where two general strategies can be adopted. The first one considers the realization of a very fast spatial variation of the SoP along the beam transverse plane. If the detector integration area is much greater than the SoP spatial variation, the resulting beam presents an effective depolarization effect. This is the case of the cholesteric LC wedge depolarizer^18^ or the LC depolarizers designed with randomly distributed optical axes^19,20^. The second strategy considers using optical modulators to generate a fast temporal variation of the SoP. In this case, if the detector integration time is much greater than the SoP temporal variation, again the result is an effective depolarization effect. This effect was noticed originally in liquid–crystal on silicon (LCOS) displays^21,22^, where it was perceived as a negative effect that reduced the image contrast or the diffraction efficiency of patterns displayed onto these devices. However, more recently it has been exploited to create depolarizer instruments based either on ferroelectric LC modulators^23^ or acousto-optic modulators^17^.

All these LC depolarizers were performed using a non-expanded laser beam and a single-pixel modulator. However spatial light modulators (SLM) are electronically controlled two-dimensional LC arrays consisting nowadays of over 1000 × 1000 pixels, and with a pixel size in the order of a few microns. Therefore, such LC-SLMs can be exploited to generate customized depolarization effects.

In this work, we use a parallel-aligned LC-SLM to emulate a temporal depolarizer in order to create an effective DoP image. We obtain this new situation by encoding a pixelated retardance pattern that changes with time. Such temporal variation has been proven to be very effective to reduce speckle noise in computer-generated holograms^24,25^. In those works, however, SLMs were exploited only as scalar phase-only devices since no variation of the SoP was produced. Here, on the contrary, we use the SLM to produce a temporal SoP variation resulting in an effective depolarization effect which, in addition, is made spatially variant.

Since there is an increasing interest in studying the depolarization indices^26^ as channels of new information, for instance in biological samples^27^, this capability of emulating the depolarization with controlled precision can help to understand the physical mechanisms that cause depolarization in these samples. In addition, systems that generate structured light often make use of vector beams^28^, where the SoP varies spatially but where the beam is usually kept totally polarized^29^. As illustrated in this work with some examples, including the DoP as a new parameter in the vector beams could lead to completely new concept of structured light designs.

The structure of the paper is as follows. After this introduction, the next section introduces the methods we have applied, including the Mueller matrix analysis of a retarder with a temporal variation of its retardance and a graphical description of the time averaged SoP and its expected depolarization characteristics. It also includes the description of the experimental system. “Results and discussion” section shows the experimental results obtained by Mueller matrix image polarimetry, which proves the expected generation of spatially-variant effective depolarizing patterns. Various cases featuring different time and spatially-varying phase patterns are considered. Their complete interpretation is provided based on the SoP variations in the Poincaré sphere. Finally, “Conclusion” section includes the conclusions of the work.

## Methods

### Time averaged Mueller matrix

A state of polarization can be described by its Stokes parameters^1^ ($S_{0}$, $S_{1}$, $S_{2}$_,_ $S_{3}$) and they are represented in a column vector ${\mathbf{S}} = \left[ {S_{0}, S_{1}, S_{2}, S_{3}} \right]^{T}$. An effective SoP that corresponds to partially polarized light (mixed state) can be described by a Stokes vector resulting from the incoherent addition of two Stokes vectors that describe fully polarized light (pure states). This addition can be experimentally performed by temporally averaging in a time $T$ two Stokes vectors describing fully polarized light (${\mathbf{S}}_{A}$ and ${\mathbf{S}}_{B}$), each weighted by the time $t_{A}$ and $t_{B} = (T - t_{A} )$, respectively, where $t_{A} \le T$^23^. As a result, an effective Stokes vector $\langle{\mathbf{S}}_{e}\rangle$ is obtained:

1 $$\langle{\mathbf{S}}_{e} \rangle= \frac{1}{T}\left[ {t_{A} {\mathbf{S}}_{A} + (T - t_{A} ){\mathbf{S}}_{B} } \right].$$

In this work, we use this principle to make polarization patterns with a controlled spatially-varying DoP. For this purpose, a LC-SLM is illuminated with polarized light and two patterned gray-level designs are sequentially addressed to the SLM and switched during a period $T$, which is taken to be the integration time of our detector. As a result, two different spatially-varying pure polarization states are generated at the output, which change in time according to Eq. (1). The resulting light pattern can be regarded as having a customized effective partially polarized SoP, provided the integration time in the detector is large enough compared to the switch time in the SLM. Let us note that this procedure can be extended to include more than two polarization states. However, as shown next, with just two states it is possible to achieve any degree of polarization, including a full depolarization.

Therefore, for simplicity, we assume the situation where $t_{A} = T/2$. Therefore, using Eq. (1), the effective Stokes vector after the SLM plane is given by:

2 $$\langle{\mathbf{S}}_{e} \left( {\mathbf{x}} \right)\rangle = \frac{1}{2}\left[ {{\mathbf{S}}_{A} \left( {\mathbf{x}} \right) + {\mathbf{S}}_{B} \left( {\mathbf{x}} \right)} \right],$$

where ${\mathbf{S}}_{A} \left( {\mathbf{x}} \right)$ and ${\mathbf{S}}_{B} \left( {\mathbf{x}} \right)$ are the Stokes vectors describing the SoP that is generated by applying pattern A and pattern B to the SLM, and where ${\mathbf{x}} = \left( {x,y} \right)$ represent the spatial coordinates in the SLM.

The transformation that the SLM performs on an arbitrary input beam with homogeneous SoP $\left( {{\mathbf{S}}_{in} } \right)$ can be described by a spatially-varying Mueller Matrix. For each of the two displayed patterns we can define its Mueller matrix ${\mathbf{M}}_{A} \left( {\mathbf{x}} \right)$ and ${\mathbf{M}}_{B} \left( {\mathbf{x}} \right)$ that apply over the input polarization state ${\mathbf{S}}_{in}$, resulting in two different polarization states: ${\mathbf{S}}_{A} \left( {\mathbf{x}} \right) = {\mathbf{M}}_{A} \left( {\mathbf{x}} \right) {\mathbf{S}}_{in}$ and ${\mathbf{S}}_{B} \left( {\mathbf{x}} \right) = {\mathbf{M}}_{B} \left( {\mathbf{x}} \right){ }{\mathbf{S}}_{in}$. Therefore, the effective averaged Mueller matrix during a time $T$ is:

3 $$\langle{\mathbf{M}}_{e} \left( {\mathbf{x}} \right)\rangle = \frac{1}{2}\left[ {{\mathbf{M}}_{A} \left( {\mathbf{x}} \right) + {\mathbf{M}}_{B} \left( {\mathbf{x}} \right)} \right].$$

The SLM used in this work is a parallel-aligned LC-SLM with its principal axis horizontally oriented. Therefore, it can be considered as a pixelated linear retarder where each pixel has the same principal axis orientation but with a variable retardance. This is represented by the Mueller matrix of a linear retarder with retardance $\phi$ and its slow axis along the horizontal direction^1^.

4 $${\mathbf{M}}_{R} \left( \phi \right) = \left( {\begin{array}{*{20}c} 1 & 0 & 0 & 0 \\ 0 & 1 & 0 & 0 \\ 0 & 0 & {\cos \phi } & { - \sin \phi } \\ 0 & 0 & {\sin \phi } & {\cos \phi } \\ \end{array} } \right).$$

Substituting Eq. (4) in (3) the effective Mueller Matrix $\langle{\mathbf{M}}_{e} \left( {\phi_{A} ,\phi_{B} } \right)\rangle$ that describes the SLM is:

5 $$\langle{\mathbf{M}}_{e} \left( {\phi_{A} ,\phi_{B} } \right) \rangle= \frac{1}{2}\left[ {{\mathbf{M}}_{R} \left( {\phi_{A} } \right) + {\mathbf{M}}_{R} \left( {\phi_{B} } \right)} \right],$$

6 $$\langle{\mathbf{M}}_{e} \left( {\phi_{A} ,\phi_{B} } \right)\rangle = \left( {\begin{array}{*{20}c} 1 & 0 & 0 & 0 \\ 0 & 1 & 0 & 0 \\ 0 & 0 & {\frac{1}{2}\left( {\cos \phi_{A} + \cos \phi_{B} } \right)} & { - \frac{1}{2}\left( {\sin \phi_{A} + \sin \phi_{B} } \right)} \\ 0 & 0 & {\frac{1}{2}\left( {\sin \phi_{A} + \sin \phi_{B} } \right)} & {\frac{1}{2}\left( {\cos \phi_{A} + \cos \phi_{B} } \right)} \\ \end{array} } \right),$$

where $\phi_{A}$ and $\phi_{B}$ are the retardance function for pattern A and pattern B encoded in the SLM during one period, and where the ${\mathbf{x}}$ dependence in these relations was omitted for simplicity.

### Mueller–Stokes transformations

The above effective Mueller matrix is analysed using the well-known Lu–Chipman decomposition^30^, which defines the Mueller matrix as the product of the Mueller matrices of a depolarizer ${\mathbf{M}}_{\Delta }$, a retarder ${\mathbf{M}}_{R}$ and a diattenuator ${\mathbf{M}}_{D}$. The Mueller matrix ${\mathbf{M}}_{\Delta }$ of a depolarizing element with its principal axes aligned along the ${\text{S}}_{1}$, ${\text{S}}_{2}$ and ${\text{S}}_{3}$ axes is given by

7 $${\mathbf{M}}_{\Delta } = \left( {\begin{array}{*{20}c} 1 & 0 & 0 & 0 \\ 0 & {a_{1} } & 0 & 0 \\ 0 & 0 & {a_{2} } & 0 \\ 0 & 0 & 0 & {a_{3} } \\ \end{array} } \right),$$

with $\left| {a_{j} } \right| = \left( {1 - \Delta_{j} } \right) \le 1$, $j = 1,2,3$, being $a_{j}$ the principal depolarization factors and $\Delta_{j}$ the depolarizance along the ${\text{S}}_{1}$, ${\text{S}}_{2}$ and ${\text{S}}_{3}$ axes^30^.

The null value of the elements of the first row and column in Eq. (6) reveals the expected result that the system does not present diattenuation nor polarizance. Therefore, the matrix $\langle{\mathbf{M}}_{e} \left( {\phi_{A} ,\phi_{B} } \right)\rangle$ can be decomposed as the product of a pure depolarizer and a linear retarder. It is thus straightforward to show that it can be written as the following product of two matrices:

8 $$\langle{\mathbf{M}}_{e} \left( {\phi_{A} ,\phi_{B} } \right)\rangle = \left( {\begin{array}{*{20}c} 1 & 0 & 0 & 0 \\ 0 & 1 & 0 & 0 \\ 0 & 0 & {\cos \overline{\delta }} & 0 \\ 0 & 0 & 0 & {\cos \overline{\delta }} \\ \end{array} } \right)\left( {\begin{array}{*{20}c} 1 & 0 & 0 & 0 \\ 0 & 1 & 0 & 0 \\ 0 & 0 & {\cos \overline{\phi }} & { - \sin \overline{\phi }} \\ 0 & 0 & {\sin \overline{\phi }} & {\cos \overline{\phi }} \\ \end{array} } \right),$$

where $\overline{\phi }$ is the arithmetic averaged retardance:

9 $$\overline{\phi } = \frac{{\phi_{A} + \phi_{B} }}{2},$$

and $\overline{\delta }$ is their semi-difference:

10 $$\overline{\delta } = \frac{{\phi_{A} - \phi_{B} }}{2}.$$

Thus, Eq. (8) shows that the effective Mueller matrix can be viewed as the combination of a linear retarder aligned along the ${\text{S}}_{1}$ axis followed by a depolarizer aligned along ${\text{S}}_{1}$, ${\text{S}}_{2}$ and ${\text{S}}_{3}$ axes.

**Figure 1:**
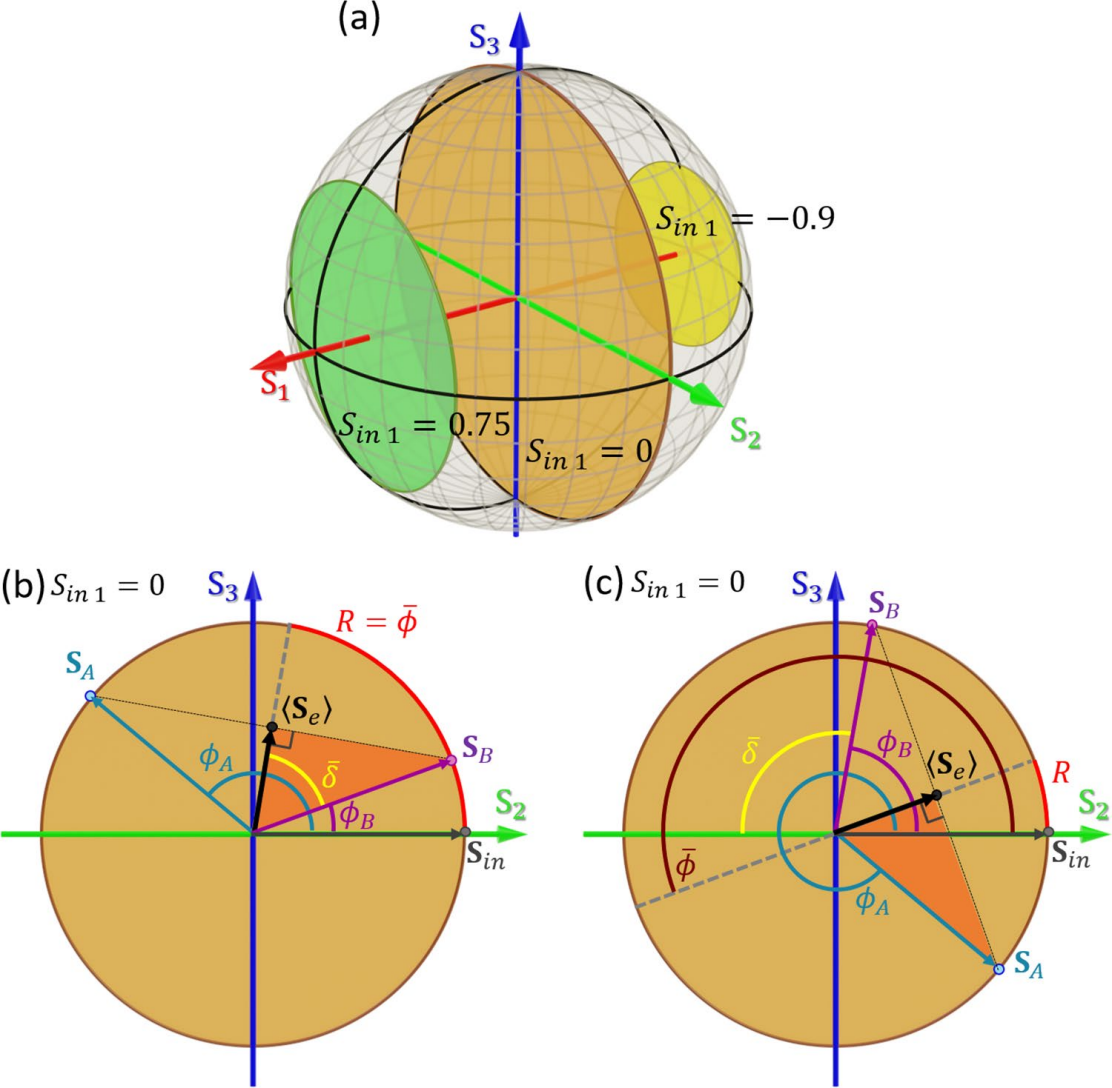
(**a**) Planes in the Poincaré Sphere defined by a constant value of ${\text{S}}_{1}$. The plane that contains the input state ${\mathbf{S}}_{in}$ defines all the possible SoPs that the effective state $\langle{\mathbf{S}}_{e}\rangle$ can reach. Note that only the plane ${\text{S}}_{1} = 0$ contains all the possible DoP values. (**b**,**c**) Composition of the effective output state $\langle{\mathbf{S}}_{e}\rangle$ as the incoherent addition of states ${\mathbf{S}}_{A}$ and ${\mathbf{S}}_{B}$ for an input state with $S_{in 1} = 0$ when $\cos \overline{\delta } > 0$ (**b**) and when $\cos \overline{\delta } < 0$ (**c**).

Figure 1 illustrates on the Poincaré Sphere (PS) the polarization changes induced by such effective Mueller matrix when acting upon an input polarization state ${\mathbf{S}}_{in} = \left[ {S_{in 0}, S_{in 1}, S_{in 2}, S_{in 3}} \right]^{T}$. Since the neutral axes of the effective retarder are along the horizontal and vertical directions (${\text{S}}_{1}$ axis), the matrix $\langle{\mathbf{M}}_{e}\rangle$ only modifies the $S_{in 2}$ and $S_{in 3}$ parameters. Therefore, the output SoP will lie in the plane of the PS defined by the constant value ${\text{S}}_{in 1}$. Figure 1a shows three different planes that contain all the possible SoPs that can be generated for three different input states with diverse values of $S_{in 1}$. As illustrated, the maximum possible number of SoPs are obtained when $S_{in 1} = 0$ (orange plane in Fig. 1a), which is the case considered in this work. Note that at the intersection of planes with the PS surface we find fully polarized states, and the center of the PS corresponds to a fully depolarized state. Any other spot in the plane describes a partially polarized state.

To avoid negative values in the depolarization factors $a_{j}$ in Eq. (7), it is rewritten as $\langle{\mathbf{M}}_{e} \left( {\phi_{A} ,\phi_{B} } \right) \rangle= {\mathbf{M}}_{\Delta } \left( {\overline{\delta }} \right)\cdot {\mathbf{M}}_{R} \left( R \right)$ with

11 $$\langle{\mathbf{M}}_{e} \left( {\phi_{A} ,\phi_{B} } \right)\rangle = \left( {\begin{array}{*{20}c} 1 & 0 & 0 & 0 \\ 0 & 1 & 0 & 0 \\ 0 & 0 & {\left| {\cos \overline{\delta }} \right|} & 0 \\ 0 & 0 & 0 & {\left| {\cos \overline{\delta }} \right|} \\ \end{array} } \right)\left( {\begin{array}{*{20}c} 1 & 0 & 0 & 0 \\ 0 & 1 & 0 & 0 \\ 0 & 0 & {\cos R} & { - \sin R} \\ 0 & 0 & {\sin R} & {\cos R} \\ \end{array} } \right),$$

where the effective retardance $R$ of the retarder matrix ${\mathbf{M}}_{R} \left( R \right)$ is now given by:

12 $$R = \left\{ {\begin{array}{ll} {\overline{\phi }} & \quad {\text{when} \, \cos \overline{\delta } > 0} \\ {\text{mod} \, 2\pi \left( {\overline{\phi } + \pi } \right)} & \quad {\text{when} \, \cos \overline{\delta } < 0} \\ \text{undefined} & \quad {\text{when} \, \cos \overline{\delta } = 0} \\ \end{array} } \right.,$$

where $R$ is defined from 0 to $2\pi$. Note that when $\cos \overline{\delta } = 0$ (situation that occurs when $\phi_{A} - \phi_{B} = \pi$) the Mueller matrix $\langle{\mathbf{M}}_{e}\rangle$ becomes a pure depolarizer (Diag[1, 1, 0, 0]) regardless of the $R$ value. In this situation the retardance is not well defined, since the matrix ${\mathbf{M}}_{\Delta } \left( {\overline{\delta }} \right)$ becomes singular and cannot be inverted, and ${\mathbf{M}}_{R} \left( R \right)$ cannot be determined.

Figure 1b,c illustrate the SoP transformation induced by the effective matrix $\langle{\mathbf{M}}_{e}\rangle$ on an input state with $S_{in 1} = 0.$ We consider two situations. In the first case $\cos \overline{\delta } > 0$, i.e., the difference $\phi_{A} - \phi_{B}$ modulo $2\pi$ between the two phases is lower than $\pi$. Figure 1b depicts the plane $S_{in 1} = 0$ of the PS and shows how the effective SoP $\langle{\mathbf{S}}_{e}\rangle$ is obtained from the incoherent addition of the two Stokes vectors (${\mathbf{S}}_{A}$ and ${\mathbf{S}}_{B}$) that result from the action of matrix $\langle{\mathbf{M}}_{e}\rangle$ over an input state ${\mathbf{S}}_{in}$. According to Eq. (2), the effective output Stokes vector $\langle{\mathbf{S}}_{e}\rangle$ is located at the midpoint on the straight line joining the two points in the PS defined by the vectors ${\mathbf{S}}_{A}$ and ${\mathbf{S}}_{B}$. As depicted in Fig. 1b, the action of the effective retarder matrix ${\mathbf{M}}_{R} \left( R \right)$ can be regarded as a counter-clockwise $R = \overline{\phi }$ rotation of the input vector ${\mathbf{S}}_{in}$ around the ${\text{S}}_{1}$ axis of the PS. In turn, the action of the effective depolarizer ${\mathbf{M}}_{\Delta } \left( {\overline{\delta }} \right)$ equally changes the length of the $S_{in 2}$ and $S_{in 3}$ parameters, and consequently it is related to the DoP of the output vector $\langle{\mathbf{S}}_{e}\rangle$.

Figure 1c illustrates the SoP transformation when $\cos \overline{\delta } < 0$, a situation that occurs when the difference $\phi_{A} - \phi_{B}$ modulo $2\pi$ is larger than $\pi$. In this case, the global minus sign in $\cos \overline{\delta }$ is equivalent to shifting the effective retardance by $\pi$ with respect to $\overline{\phi }$, i.e., $R = {\text{mod}}2{\uppi }\left( {\overline{\phi } + \pi } \right)$.

### Control of the degree of polarization

The degree of polarization is defined as ${\text{DoP}} = \left( {S_{1}^{2} + S_{2}^{2} + S_{3}^{2} } \right)^{1/2} /S_{0}$, where $0 \le {\text{DoP}} \le 1$, and it corresponds to the length of the vector $\left( {S_{1} ,S_{2} ,S_{3} } \right)/S_{0}$ in the Poincaré Sphere^1^.

While the effective retarder in Eq. (11), defined by the effective retardance $R$ in Eq. (12), describes the rotation in the PS that gives the output polarization state, the corresponding DoP is controlled by the semi-difference $\overline{\delta }$. The matrix ${\mathbf{M}}_{\Delta } \left( {\overline{\delta }} \right)$ in Eq. (11) describes a non-homogeneous depolarizer. This is an expected result since we are considering a variable retarder that is always aligned along the horizontal direction. Thus, there is no change of polarization for the horizontal/vertical components. As a consequence, the horizontal/vertical depolarizance in Eq. (7) is $\Delta_{1} = 0$, while for the $\pm \;45^{\circ}$ and circular components the depolarizance is given by^1,30^

13 $$\Delta_{2} = \Delta_{3} = 1 - \left| {\cos \overline{\delta }} \right|.$$

The action of the effective Mueller matrix $\langle{\mathbf{M}}_{e} \left( {\phi_{A} ,\phi_{B} } \right)\rangle$ on an input polarization state ${\mathbf{S}}_{in} = \left[ {S_{in 0}, S_{in 1}, S_{in 2}, S_{in 3}} \right]^{T}$ yields an effective output averaged SoP described by $\langle{\mathbf{S}}_{e} \rangle= \langle{\mathbf{M}}_{e}\rangle {\mathbf{S}}_{in}$ with the following effective Stokes parameters:

14 $$\langle{\mathbf{S}}_{e} \left( {\phi_{A} ,\phi_{B} } \right)\rangle = \left( {\begin{array}{*{20}c} {S_{in 0} } \\ {S_{in 1} } \\ {\left| {\cos \overline{\delta }} \right|\left( {S_{in 2} \cos R - S_{in 3} \sin R} \right)} \\ {\left| {\cos \overline{\delta }} \right|\left( {S_{in 2} \sin R + S_{in 3} \cos R} \right)} \\ \end{array} } \right).$$

Its effective degree of polarization (${\text{DoP}}_{{{\langle\mathbf{S}}_{e}\rangle }}$) is therefore given by:

15 $${\text{DoP}}_{{{\mathbf{S}}_{e} }} = \frac{{\sqrt {S_{in 1}^{2} + \left( {S_{in 2}^{2} + S_{in 3}^{2} } \right)\cos^{2} \overline{\delta }} }}{{S_{in 0} }}.$$

In this work, we consider input SoPs that are fully polarized, so their degree of polarization is always one: ${\text{DoP}}_{{{\mathbf{S}}_{in} }} = \left( {S_{in 1}^{2} + S_{in 2}^{2} + S_{in 3}^{2} } \right)^{1/2} /S_{in 0} = 1$. Applying this condition to Eq. (15) we obtain that the effective DoP for the average output SoP is:

16 $${\text{DoP}}_{{{\langle\mathbf{S}}_{e}\rangle }} = \sqrt {\left( {\frac{{S_{in 1} }}{{S_{in 0} }}} \right)^{2} \sin^{2} \overline{\delta } + \cos^{2} \overline{\delta }} .$$

For the case $S_{in 1} = 0$ then

17 $${\text{DoP}}_{{{\langle\mathbf{S}}_{e}\rangle }} \left( {S_{in 1} = 0} \right) = \left| {\cos \overline{\delta }} \right|.$$

These relations reveal that a total depolarization (${\text{DoP}}_{{{\langle\mathbf{S}}_{e} \rangle}} = 0$) is attained when $S_{in 1} = 0$ and $\left| {\cos {\overline{\delta }}} \right| = 0$. This happens when the states ${\mathbf{S}}_{A}$ and ${\mathbf{S}}_{B}$ lie in antipodal points of the PS (i.e., two orthogonal polarizations are added incoherently) and, consequently, $\langle{\mathbf{S}}_{e}\rangle$ is right in the center of the sphere. This situation occurs for $\overline{\delta } = \pi$/2.

### Experimental setup

**Figure 2:**
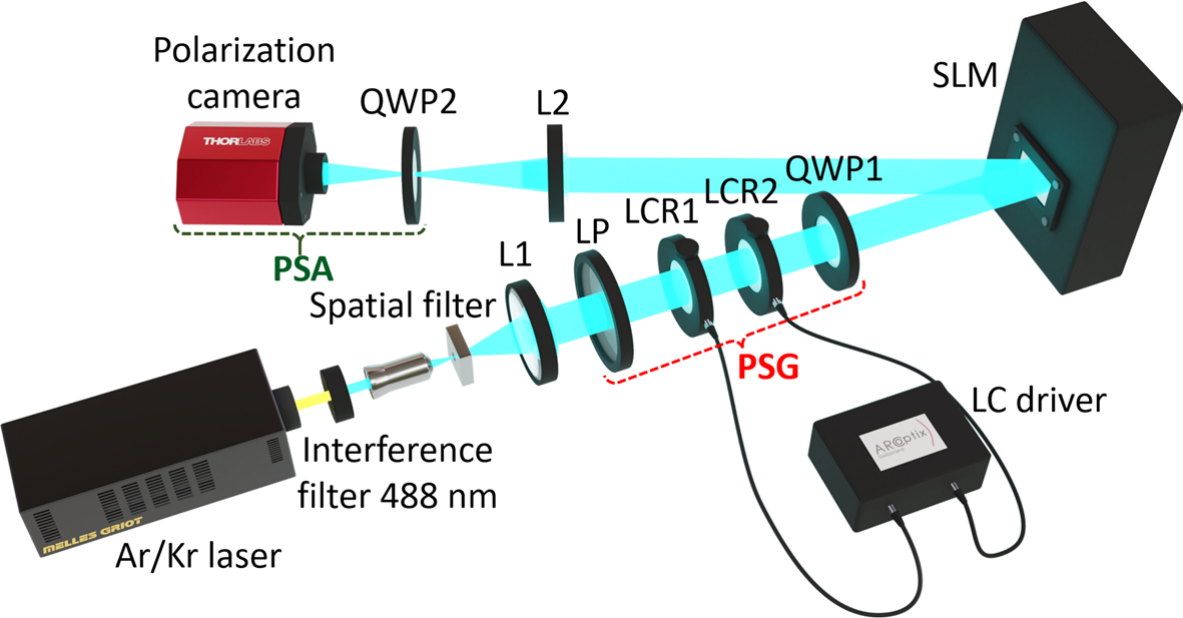
Schematic diagram of the Mueller imaging polarimeter in a reflection configuration (*L* converging lens, *LP* linear polarizer, *LCR* liquid–crystal retarder, *QWP* quarter wave-plate, *SLM* reflective spatial light modulator, *PSG* polarization state generator, *PSA* polarization state analyzer).

The experimental setup used in this work is shown in Fig. 2. It is a Mueller matrix imaging polarimeter^31^ that we have adapted to analyze the polarimetric properties of the reflective LCOS-SLM. It basically consists in two blocks: a tunable polarization state generator (PSG) based on two liquid–crystal retarders and a polarization state analyzer (PSA) based on a polarization camera.

The light source is an Argon/Krypton laser (CVI/Melles Griot, Mod. 35-LTL-835-240) whose 488 nm wavelength is selected using an interference filter. Then, the laser beam is spatially filtered and collimated before entering the liquid–crystal PSG, composed of a vertically-oriented linear polarizer followed by two commercial tunable liquid–crystal retarders (LCR) from ArcOptix^32^ with their fast axis oriented at angles of 45° and 90°, respectively. The final element of the PSG is a quarter wave-plate (QWP1, Thorlabs, AQWP05M600) with its fast axis at − 45°. This PSG is a compact version of the one given in^33^ consisting of two LCRs and two QWPs. The first LCR in Fig. 2 (LCR1) rotates the input vertical SoP around the ${\text{S}}_{2}$ axis in the Poincaré Sphere an angle equal to the selected retardance. The voltage addressed to LCR1 is set to add a $\pi /2$ retardance, resulting in an effective quarter-wave plate placed after LCR1 with its fast axis also oriented at 45°. This effective quarter-wave plate, LCR2 and QWP1 act as a polarization rotator that rotates the SoP around the ${\text{S}}_{3}$ axis an angle equals to half the retardance value selected for the second LCR. Therefore, with this PSG, the retardation values of LCR1 and LCR2 provide, respectively, the ellipticity and azimuth angles of the generated SoP. The LCR’s retardance were calibrated for the 488 nm wavelength and the generation of the standard SoPs was verified^34^.

The PSA consists in a second QWP2 with its fast axis vertically oriented and a Kiralux™ Polarization Camera (Thorlabs, CS505MUP). This camera has a monochrome CMOS sensor of 5 megapixels, with integrated four-directional wire grid polarizer array. It has macropixels of 6.9 μm consisting in four micropixels of pixel size 3.4 μm, thus making it possible to detect in a single shot the linear SoPs with orientations at 0°, ± 45° and 90°. Hence the QWP2 is added before the camera only when the circular polarizations should be detected.

In this work we use as the sample in our imaging polarimeter an LCOS-SLM (Hamamatsu X10468-01), with 800 × 600 pixels and pixel size 20 μm. This is a parallel-aligned nematic liquid–crystal on silicon display, thus acting as a reflective linear retarder where the retardance can be tuned through the gray level addressed from a computer. Because it is a reflective device the PSA arm must be placed in a reflection configuration with a reflection angle of ~ 5°. The SLM screen plane is imaged on the camera by using a second lens (L2, *f* = 200 mm) and by setting the distances to ensure a 1:3 correspondence between the SLM pixels and the camera macropixels. The SLM modulation was calibrated following standard procedures^35^ in order to obtain the correspondence of the gray scale with the retardation value for the 488 nm wavelength. A retardance variation from 0.65π to more than 4π was found for the standard one-byte gray levels ranging from 0 to 255.

The key aspect of the work is that, instead of addressing the SLM with a standard static gray-level pattern, we make the most of the real-time phase control at each pixel to address a video phase pattern that uses two different images to encode two different retardance values at each pixel. This way, the effective Mueller matrix described in Eqs. (5) and (6) can be experimentally implemented. The first image has a retardance value of $\phi_{A} \left( {\mathbf{x}} \right) = \overline{\phi }\left( {\mathbf{x}} \right) + \overline{\delta }\left( {\mathbf{x}} \right)$ and the second has $\phi_{B} \left( {\mathbf{x}} \right) = \overline{\phi }\left( {\mathbf{x}} \right) - \overline{\delta }\left( {\mathbf{x}} \right)$, where ${\mathbf{x}} = \left( {x,y} \right)$ denotes the spatial coordinates at the SLM plane.

**Figure 3:**
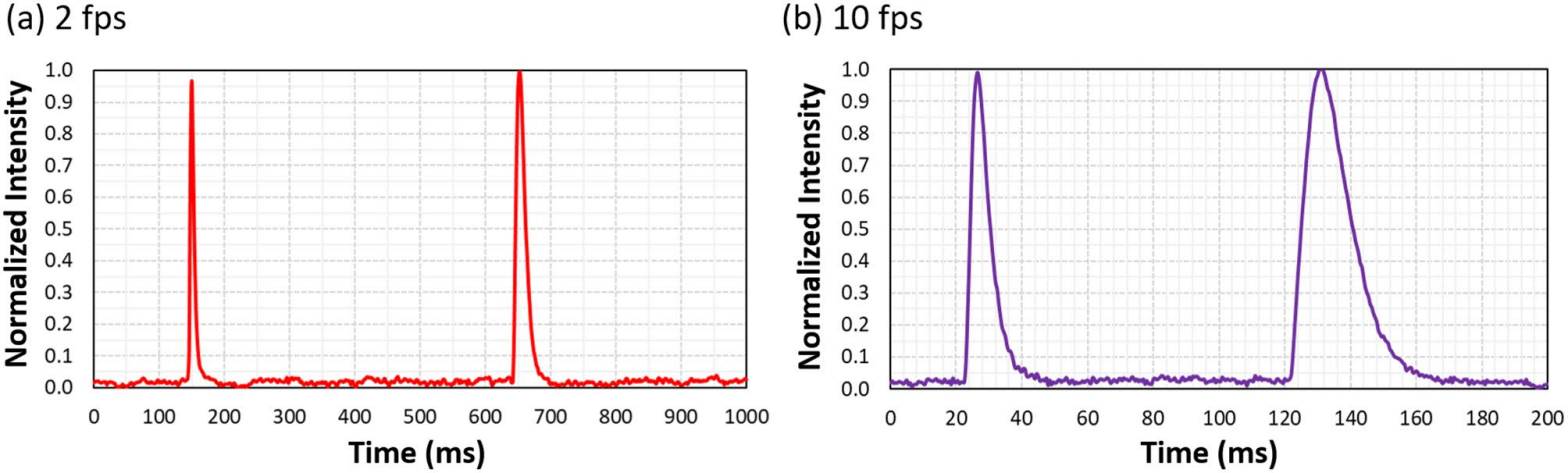
Time-resolved intensity measurements for the SLM between polarizers at $45^\circ$. Two gray levels are sequentially addressed to provide a retardance of π and 3π (**a**) every second and (**b**) every 0.2 s.

The SLM operates at video rate (60 Hz). Figure 3 shows time resolved measurements where we experimentally verified the SoP transitions that occur when frames change. We follow the experimental scheme in^21^, where the light beam reflected from the SLM is measured with a detector (Newport 818-SL) and monitored in an oscilloscope. The SLM is illuminated with linearly polarized light at $45^\circ$ with respect to the LC director. In addition, a linear polarizer is placed before the detector oriented parallel to the incident polarization. Two gray levels are sequentially addressed to the SLM, selected to provide a retardance of π and 3 π. Under this configuration, the phase difference between the gray levels is 2 π, so the same polarization state is obtained at the output. The figure shows one period of the sequence when the gray levels are switched at 2 fps (frames-per-second) and at 10 fps. Figure 3a shows how the detected signal remains constant at the expected zero intensity except for the two narrow peaks that correspond to the transitions between the two gray levels. These peaks show a different width depending on whether the gray level is increased or decreased. The sum of their widths in a period is about 60 ms, thus representing a very small fraction of the total interval when operating at 2 fps. On the contrary, when operating at 10 fps (Fig. 3b) these transitions intervals approach the period of the sequence, and therefore cannot be ignored.

Since we want to mimic the situation in Eqs. (5) and (6) with a good fidelity and reduce the impact of these transition intervals, we selected a video film with very low rate of only two frames-per-second. For higher rates, the transition intervals of the LC molecules become much more noticeable and therefore they must be considered in the calculation of the effective Mueller Matrix of the SLM. While the video was displayed on the SLM screen, a total of 36 images were captured by the camera. Each image corresponds to the generation and detection of the six standard SoPs (linear states oriented at 0°, ± 45° and, 90°, and circular states) by the LC-PSG and the PSA, respectively. Note that the use of the polarization camera reduces the number of measurements to 12 different PSG-PSA combinations. It can be argued that such a slow rate of two frames per second cannot generate a real depolarization effect. This is the reason why we name this proof-of-concept system an “emulator depolarizer system”. Although, from a theoretical point of view, depolarization is related to polarization changes at electron transitions rates, from a practical point of view, where detectors and CCDs sensors are required to conduct radiometric measurements or images, an effective depolarization would be detected. Therefore, having a detector rate even slower than the LCOS-SLM rate provides equivalent effective polarimetric results. Note that much faster response could be achieved with SLM devices based on ferroelectric liquid crystals, capable to switch at kHz rates^36^. A faster switching response can be reached with nematic liquid–crystal SLMs by applying a transient effect^37^.

The coefficients of the experimental Mueller matrix were calculated according to standard methods^38^ and the polarimeter calibration was made by measuring the air and a quarter wave plate. The use of LCR devices avoid having moving parts in the polarimeter, but when employed with a coherent source, as it is our case, induces interference fringes in the captured images. We applied a digital Notch filter to eliminate this periodic noise in the polarimetric measurements^39^.

## Results and discussion

In this section we provide the experimental results obtained with different phase pattern images that emulate samples featuring different depolarization spatial patterns. Three cases are considered: a four-quadrant pattern, a text and a spiral pattern where the SoP and DoP are spatially varying.

### CASE I: four sector patterns

**Figure 4:**
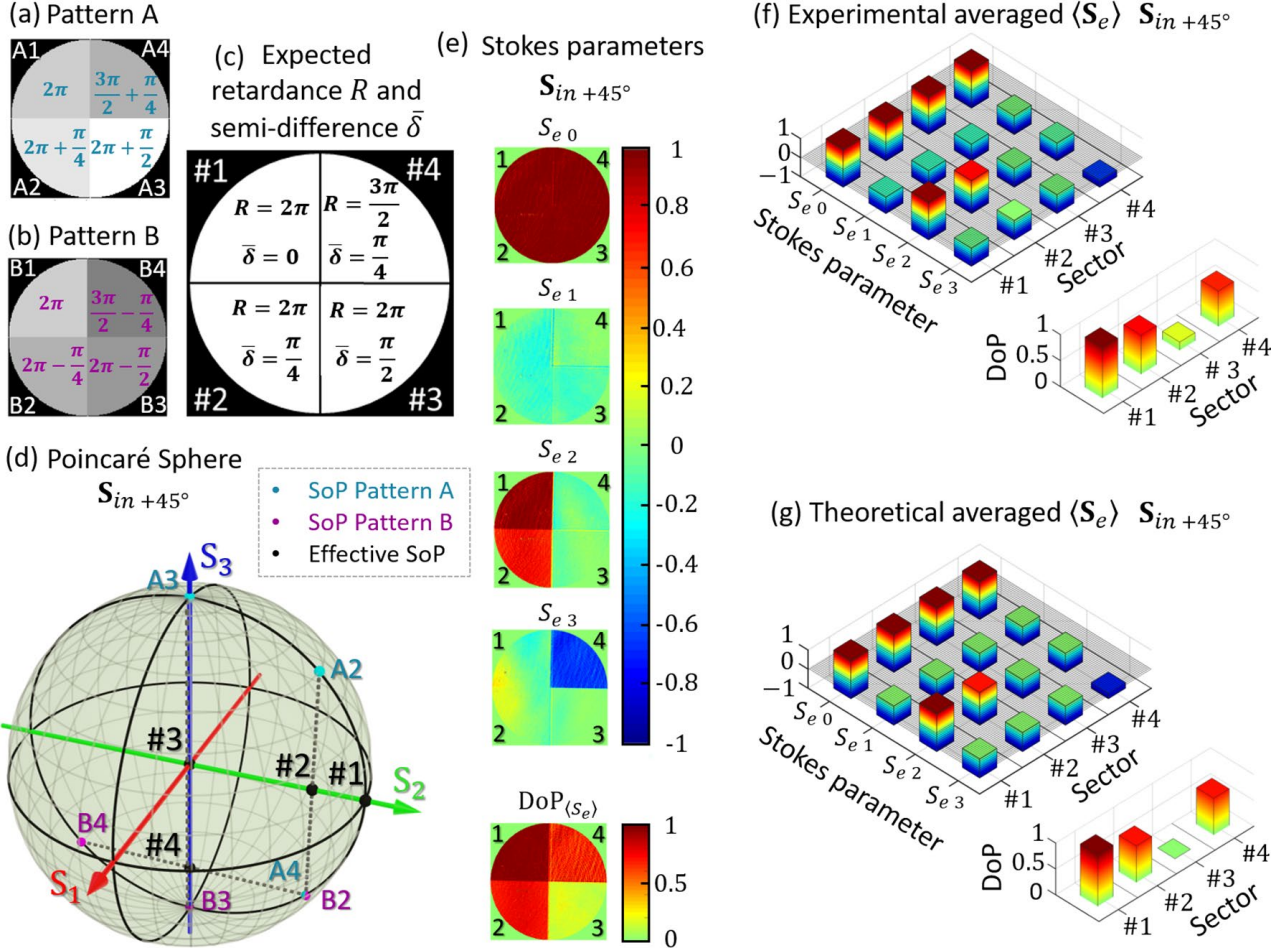
(**a**,**b**) Switching phase-pattern images. (**c**) Expected retardance and semi-difference. (**d**) Location of the theoretical effective SoP for each *j* pattern sector where $j = 1,2,3,4$ on the Poincaré sphere for a linear incident SoP at $+ \;45^\circ$. The points A-*j* and B-*j* indicate the SoP of the individual phase pattern. (**e**,**f**) Experimental output effective Stokes parameters $\langle{\mathbf{S}}_{e}\rangle = \left( {S_{e 0} ,{ }S_{e 1} ,{ }S_{e 2} ,{ }S_{e 3} } \right)$ and their respective effective ${\text{DoP}}_{{{\langle\mathbf{S}}_{e}\rangle }}$ when the incident beam is polarized at $+ 45^\circ$. (**g**) Corresponding expected theoretical Stokes parameters and DoP.

Figure 4a,b illustrate the phase pattern images $\phi_{A} \left( {\mathbf{x}} \right)$ and $\phi_{B} \left( {\mathbf{x}} \right)$, respectively. These are the two gray-level images addressed to the SLM that switch to generate the time varying retarder. In this first example they are divided into four sectors, each with a different retardance value. Sectors #A1 and #B1 lie in the upper left part of the image and the following sectors are numbered in a counter-clockwise direction. Sectors #1, #2 and #3 are designed to provide retardances around a mean value $\overline{\phi } = 2\pi$, so there is no other change than depolarization on the effective SoP compared to the input state. Figure 4c shows the expected effective retardance $R$ and the semi-difference $\overline{\delta }$.

To understand how this effective depolarization generator works, we have illustrated the output SoP for each sector separately, as well as the theoretical effective SoP on the Poincare sphere for an incident beam with + 45° linear polarization (${\mathbf{S}}_{in +45^\circ } = \left[ {1, 0, 1, 0} \right]^{T}$). Figure 4d illustrates the expected polarization transformations in the Poincaré sphere. The input state is the black point #1, which also corresponds to the output state for sector #1, thus remaining fully polarized. For the other sectors, the output states for each individual phase pattern (A and B) are indicated in the PS as points Aj and Bj, where j = 2,3,4 denotes the sector. The time-averaged output SoP is drawn as the black spots #2, #3 and #4. When the retardation difference in both phase images is $2\overline{\delta } = \pi /2$ (sectors #2 and #4), the effective SoPs lie inside the Poincaré sphere on states $\langle{\mathbf{S}}_{e,2}\rangle = \left[ {1, 0, 1 / \sqrt 2 , 0} \right]^{T}$ and $\langle{\mathbf{S}}_{e,4}\rangle = \left[ {1, 0, 0, - 1 / \sqrt 2 } \right]^{T}$ (black dots #2 and #4), therefore they have ${\text{DoP}} = 1/\sqrt 2$. On the other hand, when the input SoP is reflected by sector #3, the output individual SoP switches between right and left circular polarizations (points A3 and B3) and, consequently, the effective SoP lies in the center of the sphere, $\langle{\mathbf{S}}_{e,3}\rangle = \left[ {1, 0, 0, 0} \right]^{T}$, (black spot number #3). In this case, the generation of two individual orthogonal SoPs results in a fully depolarized averaged SoP.

Images of the experimental output effective Stokes parameters (${\mathbf{S}}_{e}$) obtained with the imaging polarimeter for an input $+$ 45$^\circ$ linear state (${\mathbf{S}}_{{in +45^{{\text{o}}} }}$) are shown in Fig. 4e, together with the corresponding measured DoP. The average parameters at each sector are presented in Fig. 4f and their theoretical values are plotted in Fig. 4g for comparison. Sectors #A1 and #B1 have the same retardance ($R = 2\pi$), therefore the Stokes parameters of the input SoP are not modified and the output effective ${\text{DoP}}_{{{\langle\mathbf{S}}_{e} \rangle}}$ approaches to 1. Sectors #2 and #4 modify the polarization of the incident SoP since the output value of $S_{e2}$ and $S_{e3}$ in Fig. 4e are no longer one, resulting in a partially polarized output with effective ${\text{DoP}}_{{{\langle\mathbf{S}}_{e} \rangle}}$ close to the expected value of $1/\sqrt 2$. Finally, the effective output Stokes parameters for sector #3 resembles that of unpolarized light. The experimental non-null parameter $S_{e1}$ makes the ${\text{DoP}}_{{{\langle\mathbf{S}}_{e}\rangle }}$ value not exactly zero; this slight discrepancy may be due to the transition intervals between frames in the LCOS-SLM or to experimental errors of the polarimeter system^31^. Nevertheless, these results demonstrate the ability of the procedure to emulate spatial patterns with variable SoP and DoP.

**Figure 5:**
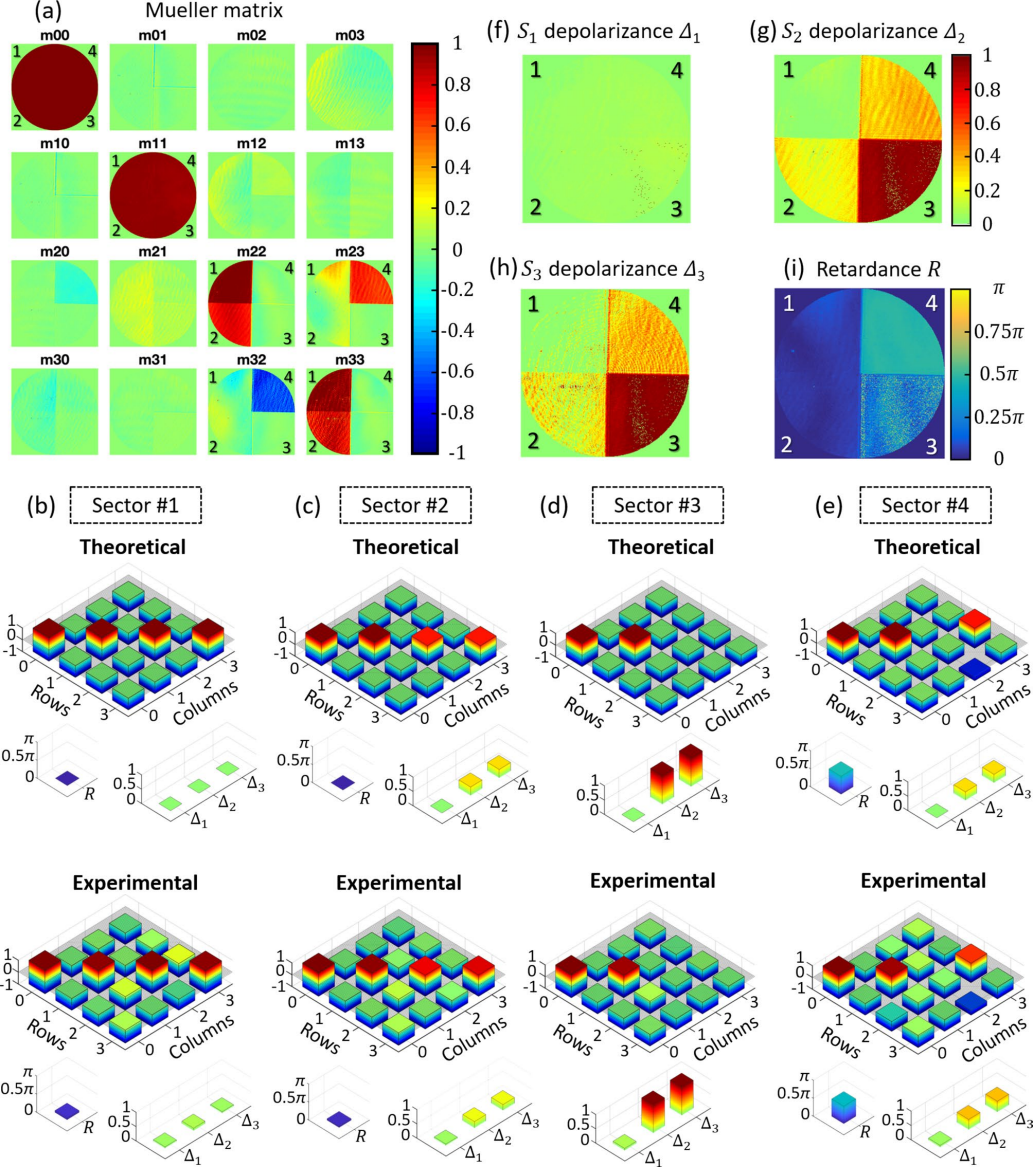
(**a**) Experimental effective Mueller matrix images for the four-sector pattern. (**b**–**e**) Comparison of the theoretical Mueller matrix averaged values. (**f**–**h**) Effective depolarizance $\left( {\Delta_{1} ,\Delta_{2} ,\Delta_{3} } \right)$ and (**i**) effective retardance ($R$).

A complete characterization of the SLM as a depolarization emulator requires obtaining its experimental Mueller matrix image. In this case, it is an effective matrix that describes the complete polarimetric response of the SLM when being addressed with time-varying patterns for any incident SoP. For that purpose, we consider the six typical SoPs (H, V, $+$ 45$^\circ$, − 45$^\circ$, RCP and LCP) in both the PSG and PSA. Figure 5a shows the experimental effective Mueller matrix $\langle{\mathbf{M}}_{e}\rangle$, normalized by the $m_{00}$ element. Figure 5a shows the images derived for the 16 elements of the Mueller matrix. The four sectors of the encoded phase patterns are only clearly visible in four elements of the lower-right 2 × 2 submatrix. These Mueller matrix elements are averaged considering all the pixels within each sector and they are plotted in Fig. 5b–e, together with the theoretical values. The corresponding numerical data are provided in Table 1. The result in all cases show a very good agreement. For instance, the effective matrix in sector #1 (Fig. 5b) resembles very well the identity matrix, $\langle{\mathbf{M}}_{e,1}\rangle \approx {\mathbf{I}}$, while in sector #2, $\langle{\mathbf{M}}_{e,2}\rangle$ becomes a diagonal matrix (Fig. 5c) with $m_{00} = m_{11} = 1$ but coefficients $m_{22}$ and $m_{33}$ reduced to $1/\sqrt 2$. A similar situation occurs in sector #3, where $\langle{\mathbf{M}}_{e,3}\rangle$ is now the diagonal matrix Diag[1, 1, 0, 0]) (Fig. 5d). Finally, in sector #4 we obtain the Mueller matrix with all elements null except $m_{00} = m_{11} = 1$, $m_{23} = 1/\sqrt 2$ and $m_{32} = - 1/\sqrt 2$.

**Table 1:**
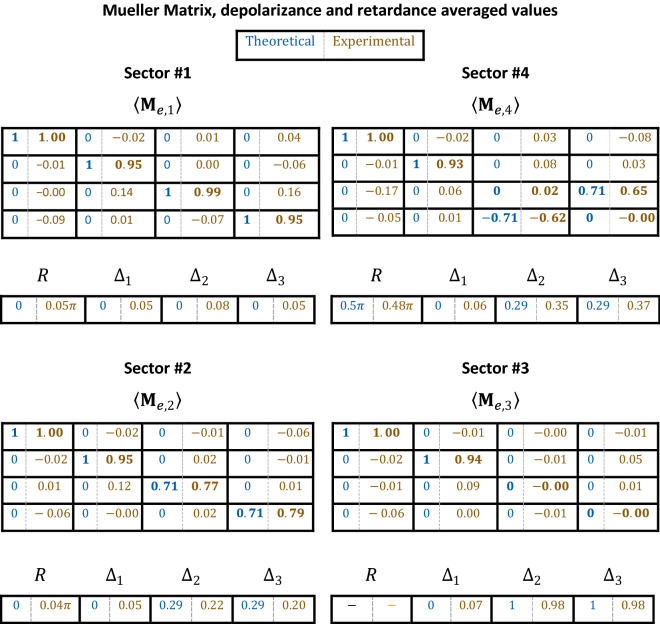
Experimental and theoretical effective Mueller matrices, depolarizance $\left( {\Delta_{1} ,\Delta_{2} ,\Delta_{3} } \right)$ and retardance ($R$) averaged values, as presented in Fig. 5.

Comparison of the theoretical and experimental data in Fig. 5 and in Table 1 show very good agreement, within the limits of the instrumental error provided by our developed imaging polarimeter. This error was estimated in^31^ using polarizers and retarders as samples, and was shown to be in all cases less than 7%. It was attributed to different error sources like the precise retardance of the LCR devices employed in the PSG, the required movable QWP in the PSA, or the speckle noise caused by laser light. In addition, slight deviations of the SLM modulation from the ideal two-phase pattern also contribute to these discrepancies. Nevertheless, these results illustrate how the temporal sequence addressed to the SLM can be used to control its effective Mueller matrix.

The Lu–Chipman decomposition^30^ was applied to the experimental effective Mueller matrix to calculate the main polarimetric parameters, like diattenuation $\left( D \right)$, polarizance $\left( P \right)$, retardance $\left( R \right)$ and depolarization ($\Delta$), as if there was no a-priori knowledge of the characteristics of the sample^31^. The elements of the first row and column of the normalized ${\mathbf{M}}_{e}$ are related to diattenuation and polarizance, respectively. These parameters are not relevant in our analysis since they are almost zero (their maximum values are $D = 0.10$ and $P = 0.18$ in sector #4). This result was expected as LCOS-SLMs are considered non-dichroic elements. Figure 5f–h shows images of the depolarizance parameters $\Delta_{1}$, $\Delta_{2} ,\Delta_{3}$. Image 4(f) shows how the H/V depolarizance parameter $\Delta_{1}$ is null for all sectors, while both ± 45° linear ($\Delta_{2 }$) and circular ($\Delta_{3}$) depolarizance change in different sectors in Fig. 5g,h, becoming maximum $\Delta_{2 } \approx \Delta_{3} \approx 1$ in sector #3. Finally, the measured effective retardance is shown in Fig. 5i. Sectors #1 and #2 show experimental values close to the expected value $R = 0$, while in sector #4 the average value is $R = 0.48\pi$, very close to the expected result $R = 0.5\pi$. In sector #3, the expected retardance is not well defined, as discussed in the previous section.

### CASE II: text pattern encoded in DoP

**Figure 6:**
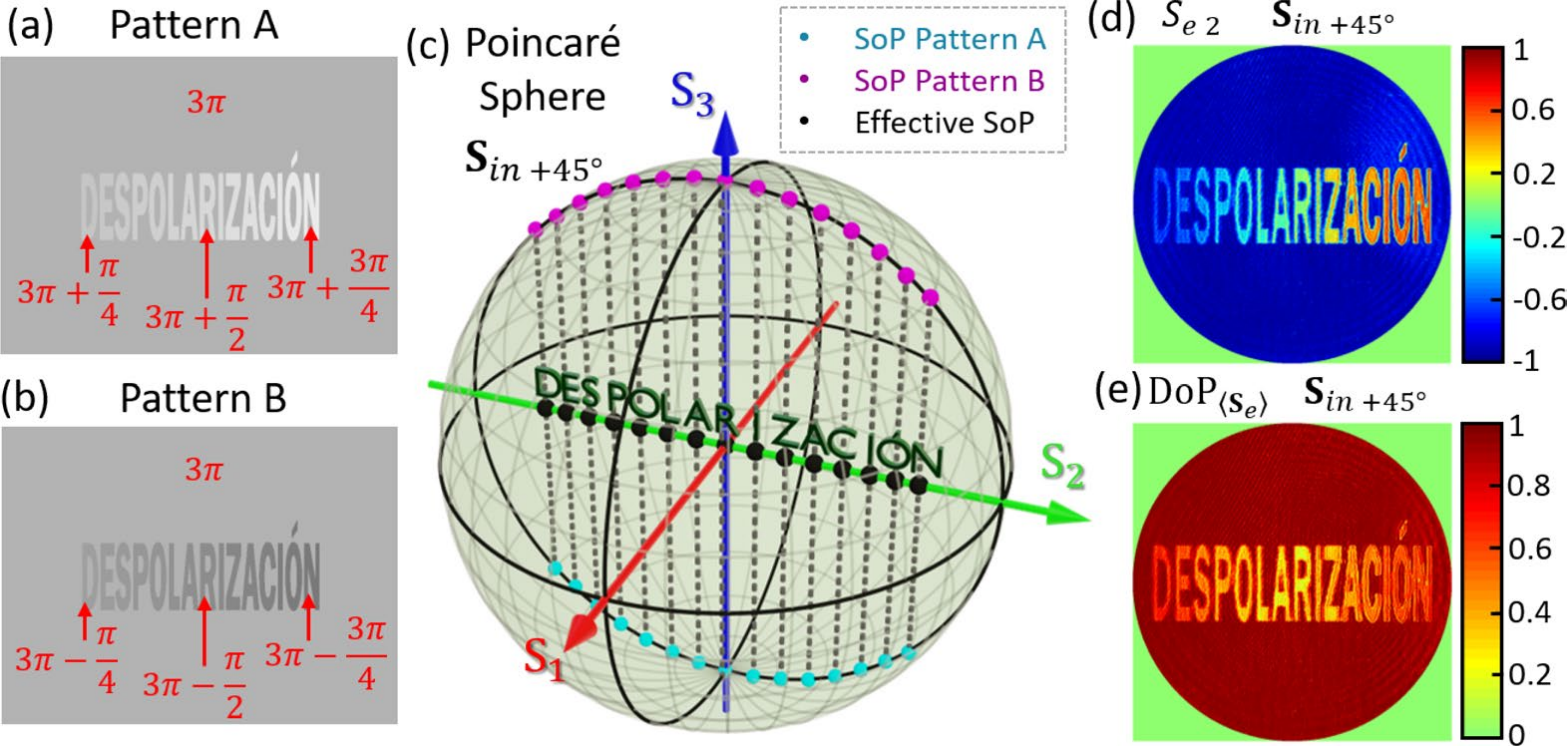
(**a**,**b**) Phase pattern images encoding a text, with indication of the phase levels, (**c**) theoretical ${\mathbf{S}}_{A}$, ${\mathbf{S}}_{B}$ and $\langle{\mathbf{S}}_{e}\rangle$ Stokes parameters on the Poincaré sphere for each letter and for an input SoP linear at $+ 45^\circ$, (**d**) experimental effective Stokes parameter $S_{e2}$ of the output beam, and (**e**) experimental effective ${\text{DoP}}_{{{\langle\mathbf{S}}_{e}\rangle }}$.

The SLM allows a full control of the retardance at every pixel. Here, we make the most of this capability in order to encode a text pattern with gradually varying depolarization. For that purpose, two phase patterns were designed, each one encoding the word DESPOLARIZACIÓN (depolarization in Spanish). Figure 6a,b show the pattern gray-level images addressed to the SLM. They encode the phase functions $\phi_{A} \left( {\mathbf{x}} \right)$ and $\phi_{B} \left( {\mathbf{x}} \right)$, where about 30 × 40 pixels were used for each letter. The phase value in some specific letters and in the background is indicated in the figure. The averaged retardance is always $\overline{\phi } = 3\pi$ and the retardance difference gradually changes from $\overline{\delta } = 3\pi /4$ to $\overline{\delta } = \pi /4$ in steps of $\pi /28$. Again, we illuminate the SLM with a fully polarized state ${\mathbf{S}}_{in +45^\circ } = \left[ {1, 0, 1, 0} \right]^{T}$. Figure 6c illustrates on the PS, and for each letter, the output states ${\mathbf{S}}_{A}$ (blue dots) and ${\mathbf{S}}_{B}$ (purple dots) generated by each pattern, and the expected averaged SoP (black dots). Note that states ${\mathbf{S}}_{A}$ and ${\mathbf{S}}_{B}$ lie on points opposite to each other with respect to the ${\text{S}}_{2}$ axis. Therefore, the averaged states lie all on the ${\text{S}}_{2}$ axis with an effective DoP that changes gradually from one letter to the next according to Eq. (17). In the extremes (letters D and N), the semi-difference phase is $\overline{\delta } = \pi /4$ and $\overline{\delta } = 3\pi /4$, hence their effective DoP is ${\text{DoP}}_{{{\langle\mathbf{S}}_{e}\rangle }} = 1/\sqrt 2$, while in the center (letter R) $\overline{\delta } = \pi /2$ and the output becomes fully depolarized, ${\text{DoP}}_{{{\langle\mathbf{S}}_{e}\rangle }} = 0$.

Figure 6d illustrates the measured effective parameter $S_{e2}$ for input $45^\circ$ linear polarization, which shows the progressive change as we move along the word. The measured effective ${\text{DoP}}_{{{\langle\mathbf{S}}_{e} \rangle}}$, plotted in Fig. 6e, displays the expected behavior: ${\text{DoP}}_{{{\langle\mathbf{S}}_{e} \rangle}}$ takes the largest value in the letters at the extremes while gradually decreases to the center, reaching the minimum value at the central letter (R).

### CASE III: spirally shaped depolarization

As a final example, we generate a DoP spiral pattern. In the same way as spiral phase patterns are basic elements in vortex and vector beam generation, which have become much popular in the last decades, it might be interesting to explore the possibility of using DoP as an additional degree of freedom in the numerous applications of such singular beams^40^.

**Figure 7:**
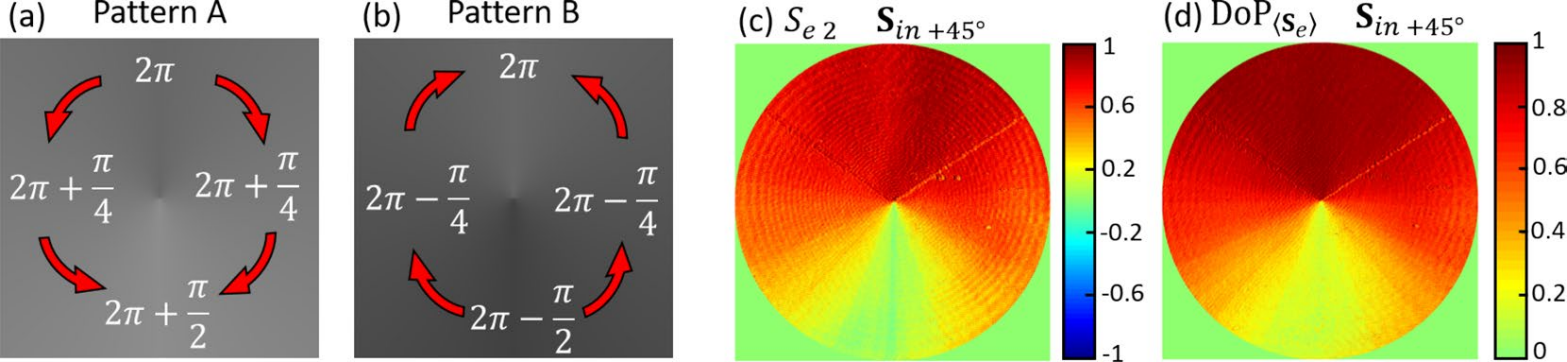
(**a**,**b**) Phase pattern images encoding a spiral distribution, with indication of the phase levels. The red arrows denote the sense where the phase increases. (**c**) Experimental effective Stokes parameter $S_{e2}$ when the incident beam is linearly polarized at $+ \;45^\circ$ and (**d**) experimental effective ${\text{DoP}}_{{{\langle\mathbf{S}}_{e} \rangle}}$ image.

To probe the effective realization of a DoP spiral pattern, we designed the two phase-patterns $\phi_{A} \left( {\mathbf{x}} \right)$ and $\phi_{B} \left( {\mathbf{x}} \right)$ shown in Fig. 7a,b, where the retardance increases azimuthally from $2\pi$ to $5\pi/2$ (starting on the y-axis) and decreases from $2\pi$ to $3\pi/2$, respectively. The averaged retardance is $\overline{\phi } = 2\pi$. Considering an input linear state at $+ \;45^\circ$, the effective Stokes parameters are $\langle{\mathbf{S}}_{e}\rangle = \left[ {1, 0, S_{e2}, 0} \right]^{T}$ where $S_{e2}$ changes azimuthally, as demonstrated experimentally in Fig. 7c. As it is observed, ${\text{S}}_{e2} \approx 1$ at the top of the image and continuously decreases with the azimuth angle, reaching $S_{e2} \approx 0$ at the opposite direction. The experimental effective ${\text{DoP}}$ in this case coincides with the $S_{e2}$ parameter, presenting a depolarization azimuthally variant-pattern, as shown in Fig. 7d. The depolarizing pattern shows a maximum ${\text{DoP}}_{{{\langle\mathbf{S}}_{e}\rangle }} \approx 1$ at the top of the image, where $\overline{\delta }$ is zero, and a minimum ${\text{DoP}}_{{{\langle\mathbf{S}}_{e} \rangle}} \approx 0$ at the bottom of the image, where $\overline{\delta } = \pi /2$. Thus, at the center there is a polarization singularity, in this case encoded in the ${\text{ DoP}}$ function.

## Conclusion

In summary, we have demonstrated a spatially controlled depolarization emulator based on a LCOS-SLM that is addressed with a time-varying gray level pattern, thus encoding a time-varying pixelated linear retarder. This allows us to perform spatial light patterns where both the state of polarization and the degree of polarization can be controlled at will.

As a proof-of-concept, three depolarization spatial patterns are realized. We name the system a “depolarization emulator” because the LCOS-SLM operates at very low rate; hence, large integration times are required in the polarimetric procedure. Nevertheless, the experimental results in this proof-of-concept demonstrate the realization of spatially varying light patterns with controlled DoP, and equivalent results could be obtained with faster SLMs, like ferroelectric liquid–crystal devices.

We describe the polarization transformations for a linear retarder depolarizer consisting in a two-level retardance time-varying pattern. An analysis of the time averaged Mueller matrix and its implications on the Poincaré sphere transformations is provided. We have shown that the output effective polarization state is governed by the averaged retardance $\overline{\phi }$, while the degree of polarization is governed by the retardance semi-difference $\overline{\delta }$.

Finally, we have shown the generation of different spatial patterns with a controlled variation of the DoP (a four-sector pattern, a text, and a spiral pattern). The polarization properties of the output light beam were verified by imaging the SLM screen onto a polarizing camera and applying a complete Mueller matrix imaging polarimetry procedure. In all cases the measured polarization parameters agree very well with the expected results.

While spatial incoherent coupling depolarization methods are very effective to depolarize a single beam, the proposed technique allows applying a different temporal depolarization effect in different pixels, thus generating different DoP at different points of a given sample. This is a unique characteristic that could not be accomplished with a spatial incoherent depolarizer. It might be interesting for instance in testing imaging polarimeters as well as in applications where a controlled depolarization is needed, especially in situations where a spatial pattern or image is required. The system can also be very relevant in investigating the different origins of depolarization and its relationship with the different depolarization parameters.
